# Five Pond-centred Outbreaks of Cholera in Villages of West Bengal, India: Evidence for Focused Interventions

**DOI:** 10.3329/jhpn.v29i5.8895

**Published:** 2011-10

**Authors:** Rita Mukherjee, Debasish Halder, Subhasish Saha, Rudra Shyamali, Chakrabarti Subhranshu, R. Ramakrishnan, Manoj V. Murhekar, Yvan J. Hutin

**Affiliations:** ^1^Field Epidemiology Training Programme, National Institute of Epidemiology, Chennai, Tamil Nadu, India; ^2^West Bengal Health Services, Kolkata, West Bengal, India; ^3^WHO India Office, New Delhi, India

**Keywords:** Case-control studies, Cholera, Cohort studies, Cross-sectional studies, Disease outbreaks, Environment, Hygiene, Pond, Retrospective studies, Sanitation, Water pollution, India

## Abstract

In rural West Bengal, outbreaks of cholera are often centred around ponds that is a feature of the environment. Five investigations of laboratory-confirmed, pond-centred outbreaks of cholera were reviewed. Case-control odds ratios were approximated with relative risks (RRs) as the incidence was low. The environment was investigated to understand how the pond(s) could have become contaminated and could have infected villagers. The five outbreaks of cholera in 2004-2008 led to 277 cases and three deaths (median attack rate: 51/1,000 people; case fatality: 1.1%; median age of case-patients: 22 years; median duration: 13 days, range: 6-15 days). Factors significantly (p<0.05) associated with cholera in the case-control (n=4) and cohort investigations (n=1) included washing utensils in ponds (4 outbreaks of cholera, RR range: 6-12), bathing (3 outbreaks of cholera, RR range: 3.5-9.3), and exposure to pond water, including drinking (2 outbreaks of cholera, RR range: 2.1-3.2), mouth washing (1 outbreak of cholera, RR: 4.8), and cooking (1 outbreak of cholera, RR: 3.0). Initial case-patients contaminated ponds through washing soiled clothes (n=4) or defaecation (n=1). Ubiquitous ponds used for many purposes transmit cholera in West Bengal. Focused health education, hygiene, and sanitation must protect villagers, particularly following the occurrence of an index case in a village that has ponds.

## INTRODUCTION

Environmental contamination plays a key role in the dynamics of cholera. *Vibrio cholerae* inhabits seas, estuaries, brackish waters, rivers, and ponds of coastal areas of the tropical world (1). Surface water in proximity to persons with infection due to *V. cholerae* is frequently contaminated with the agent (2). A surveillance study in the neighbourhood of a culture-confirmed cholera index case-patient in Bangladesh reported that 44% of surface-water sources were positive for *V. cholerae* whereas only 2% were positive in unaffected neighbourhoods (3). *V. cholerae* survives longer in water with high salinity and those with high concentration of organic nutrients, e.g. sewage (4). In some endemic regions, virulent toxigenic cholera vibrios were recovered from surface waters (5).

Cholera is endemic in the Ganges and Brahmaputra river deltas where six of the seven recorded pandemics originated. Sewage discharges are located near water sources used for washing, bathing, drinking, and defaecation (6). These conditions perpetuate transmission. Migration of humans continues to shape the emergence, frequency, and spread of infections in geographic areas and popu-lations (7). The Ganges and Brahmaputra river deltas are densely populated. This high density of population strains the existing sanitation systems, putting people at risk for many diseases, including cholera (8,9). Planktonic copepods in surface water also play a role in the multiplication, survival, and potential transmission of *V. cholerae* (10). In Bangladesh, outbreaks of cholera almost always followed a plankton bloom (11). Human-induced climate change may be creating favourable conditions for the reproduction of the bacterium in terms of water temperature, nutrient concentration, and plankton production. Hence, it might contribute to the endemicity of cholera.

In the southern part of Indian state of West Bengal, many villages are located in a specific ecosystem characterized by the omnipresence of ponds. People from lower socioeconomic status build their houses around ponds because of the low cost of land and the availability of water. Only 9.5% of the rural population of West Bengal has access to piped drinking-water. Hence, ponds are the most convenient water source. The water of these ponds is used for many domestic purposes, e.g. bathing, cleaning kitchen utensils, and even occasionally drinking. In such villages, tubewells meant for drinking-water are often built beside ponds. Improper maintenance of these tubewells often results in mixing of pond water and drinking-water.

People often construct temporary latrines near the edges of ponds, or holes are dug for convenience. These holes are usually uncovered, and in the rainy season, faeces overflow and contaminate pond water. Ponds also get contaminated through indiscriminate defaecation, excreta disposal, or washing of faecally-soiled clothes. Of 10 outbreaks of chole-ra investigated during 2004-2008 by the Indian Field Epidemiology Training Programme (FETP) in the state of West Bengal, five (50%) were rural outbreaks geographically centred around ponds. Practices, including bathing, washing utensils and clothes, and washing mouth in pond water, were significantly associated with illness. While these factors allow generating hypothesis, they draw an incomplete picture as which factors transmit chole-ra around ponds in this setting. We reviewed these outbreaks to understand the common risk factors that could shed light on the specific mechanisms that may explain the regular occurrence of pond-centred outbreaks of cholera.

## MATERIALS AND METHODS

### Descriptive epidemiology

We reviewed abstracts and investigation reports of the Indian FETP to identify the pond-centred, laboratory-confirmed outbreaks of cholera investigated using descriptive and analytical epidemiology in villages of West Bengal. Cholera was defined as per the guidelines of the World Health Organization (WHO) (12). Cases were searched actively from door to door. We divided the number of cases by the population-size to calculate attack rates. We divided the number of deaths by the number of cases to calculate case fatality. The epidemic curves were reviewed to calculate the duration of the outbreaks and described their pattern. We also reviewed the spatial distribution of cases to count the number of ponds involved. Hypotheses were generated using time, place, and person characteristics before testing these hypotheses with analytical epidemiological investigations.

### Analytical epidemiology

Case-control and retrospective cohort investigations were conducted to identify the risk factors associated with cholera. We defined a case according to the guidelines of the WHO (12). Data were collected on the demographic characteristics, date of onset, signs, symptoms, outcome, and exposure to all possible risk factors, including various practices with respect to the use of pond water. We defined the referent exposure period as 2-5 days before the appearance of clinical signs and symptoms. Health personnel were trained to conduct interviews using a structured, standardized, pretested questionnaire written in local Bangla language. In this case-control study, we initially calculated the sample-size. We selected control against each case matched by age, sex, and neighbourhood to eliminate confounding. Then, we calculated odds ratio by comparing the discordant pairs, i.e. exposed cases, unexposed controls and exposed controls, unexposed cases.

In cohort study, we included all 121 residents of the slum and interviewed them regarding their possible exposure to risk factors, including practices relating to the use of pond water. The relative risk was calculated by comparing the incidence of disease among the exposed and the non-exposed to water sources.

Data were analyzed using the Epi Info 2005 software (CDC, Atlanta, GA, USA).

Odds ratio (OR) and relative risks (RRs) were calculated to examine the association between potential exposures and cholera. We approximated from case-control studies with RRs as the incidence was low, i.e. <5%.

### Laboratory investigations

Stool specimens were collected from the case-patients and sent to the National Institute of Cholera and Enteric Diseases and the School of Tropical Medicine, Kolkata, West Bengal. We also collected water specimens for microbiological investigations in one outbreak.

### Environmental survey

In addition to the review of the outbreak investigation report, we studied baseline practices in an area in the absence of a current outbreak. This cross-sectional survey was conducted in a cluster sample of the villages of Nadia district that had ponds. The objectives were to understand how the pond(s) could have become contaminated, and how the pond(s) could contaminate villagers.

The cluster-sampling technique was adopted to select villages with ponds of Nadia district. We first selected village-clusters with a probability proportional to the population-size and then selected an identical number of households in each village-cluster. The sample-size was calculated using the Right size software (CDC, Atlanta, GA, USA). With an estimated (50%) prevalence of exposure (as the proportion was not known), a confidence coefficient interval of 95%, an α error of 5%, and a rate of homogeneity in clusters of 0.02, we needed 27 clusters with a cluster-size of 20. We anticipated a proportion of non-response of 10%, and the sample-size became 594. In each of the 27 village-clusters, we randomly selected 20 households in which we interviewed one adult picked at random. Data were collected on sociodemographic characteristics, practices that might have contaminated ponds, and practices that might have exposed people to faecally-contaminated pond water. We estimated the prevalence of practices that could contaminate ponds and that could infect people.

### Ethical approval

The Ethical Committee of the National Institute of Epidemiology, Chennai, India, approved the study protocol for the environmental assessment in Nadia district. The investigations of the outbreaks were part of emergency responses to public-health emergencies and were covered by normal practice.

## RESULTS

### Descriptive epidemiology

**Fig. 1.**
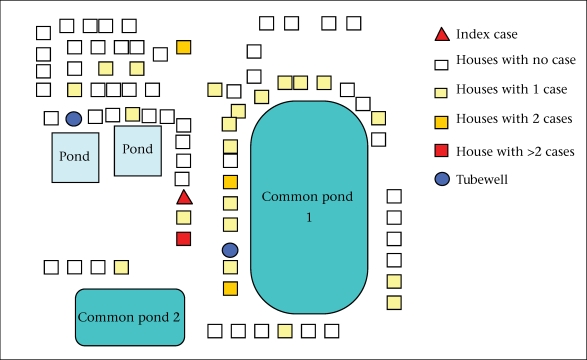
Example of clustering of cholera cases around a pond, Pipulhat village, West Bengal, 2006 (outbreak no. 4)

**Fig. 2.**
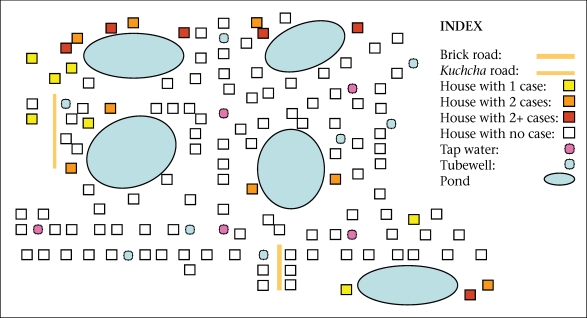
Example of clustering of cholera cases around ponds, Kachua village, West Bengal, 2004 (outbreak no. 1)

**Fig. 3.**
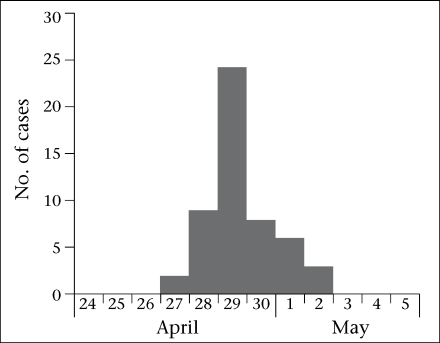
Example of point-source outbreak on an epidemic curve showing the distribution of cases by date of onset, Sonakhali village, West Bengal, 2006 (outbreak no. 2)

We reviewed five laboratory-confirmed pond-centred outbreaks of cholera. One outbreak occurred in 2004, one in 2006, two in 2007, and one in 2008. Four outbreaks occurred in a rural setting and one in a periurban setting. The five outbreaks led to 277 cases, including three deaths (case fatality: 1.1%). The median attack rate was 51 per 1,000 people. The median duration was 13 (range 6-15) days. Four outbreaks occurred during April-July and one in November. Cases were clustered around one pond in three outbreaks, around two ponds in one outbreak, and around three ponds in one outbreak (Fig. 1 and 2). The median age of the case-patients was 22 years. The epidemic curve suggested a continuous source in four occurrences (one example presented as Fig. 3), and one had a narrower peak, suggesting a point-source (Fig. 2).

### Analytical epidemiology

The analytical studies included four case-control investigations (sample-size: 55, 63, 32, and 71) and one retrospective cohort investigation (sample-size: 56). Three of the four outbreaks used a control-to-case ratio of 1:1, and one used 1:2. Factors significantly (p<0.05) associated with cholera in the studies included washing utensils in ponds, accidental swallowing of pond-water during bathing, and direct exposure to pond water, i.e. drinking, mouth washing, and cooking (Table 1). Washing utensils was associated with cholera in four outbreaks (RR: 6-12). Swallowing water during bathing was associa-ted with cholera in three outbreaks (RR: 3.5-9.3). The other group of risk factors identified was direct exposure to pond water, including drinking (two outbreaks, RR: 2.1-3.2), mouth washing (one outbreak, RR: 4.8,) and cooking with pond water (one outbreak, RR: 3.0). One study identified a dose-response relationship between swallowing water while bathing and illness, with OR 1 in the absence of exposure (never swallowed water during bath), 2.5 for 3-4 times per week, and 7.2 for a daily exposure. Similarly, while the reference OR was 1 for never rinsing mouth with pond water, the OR was 1.2 for 3-4 rinses per week and 5.2 for a daily exposure. Initial case-patients contaminated ponds through washing soiled clothes in it in four outbreaks and defaecation in one outbreak.

### Environmental survey

We approached 594 persons for participation in the survey (Table 2). All of them (100%) agreed to participate. Their median age was 32 (range 17-65) years, and 345 (58%) were males. The participants reported a number of practices that could have contaminated ponds. In total, 516 (87%) persons reported washing faecally-soiled clothes in ponds, and 496 (84%) reported disposing excreta in and around ponds. The sewerage line drained sewerage directly in the pond in 339 households (57%), and 219 persons (37%) reported using pond water to wash their body parts after defaecation. In addition, practices which could have exposed people to contaminated pond water included washing utensils in pond (n=502, 85%), washing mouth with pond water (n=439, 74%), swallowing pond water during bathing (n=438, 74%), cooking with pond water (n=380, 64%), and drinking (n=108, 18%).

## DISCUSSION

Drinking pond water was a risk factor for cholera in two of the five outbreaks. This risk factor was easily understandable from a biological point of view. A study conducted in Kenya reported an association between drinking surface water and cholera (13). However, during our survey, this practice was infrequent, with only 18% of the people reporting it. In fact, people usually collected drinking-water from taps or tubewells supplied by the local authority. Sometimes, people had no other option but to drink pond water because of an inadequate or insufficient supply of better-quality drinking-water.

Washing utensils was associated with cholera in four of the five outbreaks. The results of environmental assessment suggested that this exposure was common: 85% of the participants reported eating food in utensils washed in pond water. The poor accessibility to piped water compelled people to use pond water for these practices. As people ate food in utensils that had been washed in pond, some residual water may have been present in them. The presence of small quantities of contaminated water in containers used for eating and cooking could have inoculated uncooked food preparation that would have constituted a culture medium. Water collected from utensils in a household of a case-patient during one outbreak yielded *V. cholerae* in culture that was of the same serotype and biotype than the one isolated from a patient. This finding could support our hypothesis. However, it could also have reflected an environmental contamination in the household of a case-patient excreting *V. cholerae* during the diarrhoeal episode. A survey examined the association between self-reported past incidences of enteric diseases according to self-reported exposures to water from the Ganges river among residents of Varanasi, Uttar Pradesh, India. Logistic regression analysis indicated significant associations between enteric diseases, including cholera and use of the river water for bathing, laundering, washing cooking utensils, and brushing teeth. Thirty-three cholera cases were identified among families exposed to laundering or bathing in the Ganges river while no cholera cases occurred among unexposed families (14). Cooking food with pond water was also associated with cholera during one outbreak. While the process of cooking as practised in India would involve heating that would destroy *V. cholerae*, there might have been situations during which food preparation led to consumption of raw or incompletely-cooked food. Hence, this could have led to infections through a mechanism similar to the one we suspected for cooking utensils.

**Fig. 4.**
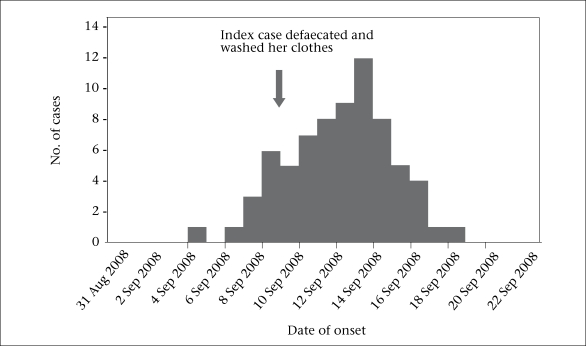
Example of continuous, common-source outbreak on an epidemic curve showing the distribution of cases by date of onset, West Bengal, 2008 (outbreak no. 5)

**Table 1.** Main epidemiological findings for pond-centred cholera outbreaks, FETP, West Bengal, India, 2004-2008

Background information	No. (year)	1 (2004)	2 (2006)	3 (2007)	4 (2007)	5 (2008)
	Setting	Rural	Urban slum	Rural	Rural	Rural
	District	South 24 Paraganas	South 24 Paraganas	South 24 Paraganas	Howrah	Nadia
Magnitude and severity	No. of cases	55	56	32	63	71
	Rate per 1,000	42	121	51	22	77
	deaths/case fatality	2 (3.6%)	0 (0%)	1 (3.1%)	0 (0%)	0 (0%)
Descriptive epidemiology	Median age (years)	18	24	22	30	20
	% of females	50	55	50	54	59
	Duration (days)	11	6	11	15	13
	No. of modes on epidemic curve	1 (common source)	1 (point source)	1 (common source)	2 (common source)	1 (common source)
	No. of ponds	3	1	1	1	2
Analytical epidemiology methods		Case control	Retrospective cohort	Case control	Case control	Case control
Relative risk /odds ratio (95% CI)	Washing utensils	N/A	12 (4.8-31)	10 (7.0-13)	9.3 (2.8-31)	6 (2.3-15)
	Swallowing water	8.3 (2.4-35)	N/A	3.5 (1.2-10)	1.3 (0.6-2.8)	9.3 (3.3-26)
	Washing mouth	N/A	N/A	N/A	1.81 (0.9-3.8)	4.8 (1.8-13)
	Drinking-water	N/A	N/A	N/A	2.1 (0.9-4.7)	3.2 (1.3-8.2)
	Cooking with water	N/A	N/A	N/A	3.0 (1.3-7.1)	0.78 (0.4-1.6)

NA=Not available;

CI=Confidence interval;

FETP=Field Epidemiology Training Programme

**Table 2.** Prevalence of practices among surveyed population residing in a 50-metre radius of ponds in Nadia district, West Bengal, 2008 (n=594)

Category	Practice	No.	%
Behaviours that could contaminate the pond	Disposed children excreta in pond	496	84
	Washed soiled clothes of a sick person in pond	516	87
	Had sewerage line draining directly into pond	339	57
	Washed body parts in pond after defaecation	219	37
Behaviours that could lead to infection of human from pond water	Washed utensils in pond	502	85
	Washed mouth with pond water	439	74
	Swallowed water during bathing	438	74
	Cooked with pond water	380	64
	Drank pond water	108	18

Washing mouth with pond water was associated with cholera in one of the five outbreaks. While people did not drink water intentionally, they might have swallowed pond water while rinsing their mouth or brushing their teeth. The study in Varanasi reported a significant association between brushing teeth with water from the Ganges river and enteric infections, including cholera (14).

Two of the five outbreaks identified bathing as a risk factor for cholera. The association of bathing with cholera is compatible with the notion that ingestion of water while bathing may contribute to the transmission of cholera (3). Thus, people may have inadvertently rinsed their mouth and swallowed pond water while taking baths. An association between bathing and cholera was also reported in a number of studies in southern Tanzania and in Burundi (15,16).

During our survey, we identified a substantial proportion of people, who reported behaviours that could have exposed the pond to faecal contamination. Those included washing faecally-soiled clothes, washing their body parts after defaecation, and disposing excreta. Of people who have a latrine in their house in rural West Bengal, 75% constructed it near the edges of pond with dug holes to accumulate faeces. In the rainy season, most of these uncovered holes overflow and contaminate surface waters. In 57% of the households surveyed, the sewerage line drained directly in pond as no closed septic system was available for those latrines.

### Limitations

Our study had three main limitations. First, we limited our report to a review of outbreak investigations with laboratory confirmation and a survey of practices. We did not conduct exhaustive microbiological assessments of the environment. We did not collect multiple laboratory specimens for the various vehicles, i.e. food sample, that we may have suspected. Hence, we can only point to behaviours that may be at risk, and we are limited in our capacity to provide detailed explanations about the biological plausibility of the hypotheses we examined. Second, the investigations were carried out in those districts where the FETP scholars were assigned. While the Indian FETP has a strong presence in the state of West Bengal (23 scholars for six districts between 2003 and 2007), our report failed to represent the regions of the state where no scholars were present and where pond-centred outbreaks of cholera might also have occurred. Third, we did not examine all the risk factors for all the outbreaks. Hence, the quantification of the number of outbreaks that identified the risk factors is an imperfect reflection of the reality.

### Conclusions

In rural West Bengal, outbreaks of cholera were often centred around ponds. Domestic and personal exposures to pond waters, such as washing cooking utensils, bathing, washing mouth, cooking, and drinking, were significantly associated with cholera. Some common practices may faecally contaminate pond while others may infect people.

### Recommendations

Based on the findings of the study, we can formulate recommendations. First, we need to educate villagers regarding the danger of pond waters that are a regular source of infection. As blocking the access to pond is impractical, we could suggest bringing the pond water to use in a separate place to wash faecally-soiled clothes or to wash body parts after defaecation. Second, we would need to build flush latrine in every household. Third, we must educate women in the community to prepare and serve food in dry utensils or in utensil washed again with clean water. Finally, we must educate people to minimize the practice that may lead to swallowing of pond water during bathing or mouth washing. For mouth washing, people could filter the water with a piece of *sari* (cloth). A study in Bangladesh has shown that this procedure was effective (17). In the longer term, improvement of the infrastructure would be needed in terms of housing, water supply, and sanitation to place populations in safer conditions with respect to these settings that seem to create vicious cycles of contamination between the humans and their environment. Finally, the number of risk factors involved and the omnipresence of the pathogen provide a rationale to examine future options to immunize the at-risk population against cholera (18).

## ACKNOWLEDGEMENTS

The study was supported by the Department of Health, Government of West Bengal, India and Government of India (Indian Council of Medical Research).

The authors thank Dr. Dipika Sur, senior epidemiologist, National Institute of Cholera and Enteric Diseases, Kolkata, West Bengal, India, for comments and suggestions throughout this project and on the manuscript.
